# Trends in Tumor Site-Specific Survival of Bone Sarcomas from 1980 to 2018: A Surveillance, Epidemiology and End Results-Based Study

**DOI:** 10.3390/cancers13215381

**Published:** 2021-10-27

**Authors:** Xianglin Hu, Kai Deng, Hui Ye, Zhengwang Sun, Wending Huang, Yangbai Sun, Wangjun Yan

**Affiliations:** 1 Department of Musculoskeletal Surgery, Fudan University Shanghai Cancer Center, Shanghai 200032, China; 17211210008@fudan.edu.cn (X.H.); zhengwangsun1985@gmail.com (Z.S.); drhuangwending@gmail.com (W.H.); 2 Department of Oncology, Shanghai Medical College, Fudan University, Shanghai 200032, China; 3 Department of Orthopedic Surgery, Affiliated Traditional Chinese Medicine Hospital of Southwest Medical University, Luzhou 646000, China; dengkaiswmu21@gmail.com; 4 Simmons Comprehensive Cancer Center, University of Texas Southwestern Medical Center, Dallas, TX 75390, USA; hui.ye@utsouthwestern.edu

**Keywords:** primary malignant bone and joint tumors, bone cancer, sarcoma, survival, time trend

## Abstract

**Simple Summary:**

Bone sarcomas are rare cancers coming from the mesenchyme cells of the body skeletal system. Osteosarcoma, chondrosarcoma, Ewing sarcoma, and chordoma are the four familiar subtypes. They may occur in any site of the whole body, bones and joints, causing disability and high fatality. Over the past four decades, many common cancers have persistently improved survival. However, as rare cancers, bone sarcomas receive less attention; it remains unknown whether bone sarcomas survival has improved. In this population-based big-data study, we explored bone sarcomas survival trends across 39 years. We found that bone sarcomas at pelvic bones, sacrum, coccyx and associated joints, as well as vertebral column continue to have high mortality risks. Bone sarcomas survival significantly improved in the 1990s, however, it stopped further improving during the latest three decades. More trials regarding cancer immune and targeted therapy are needed in bone sarcomas individuals to improve survival.

**Abstract:**

Objectives: As diagnosis and treatment guidelines for bone sarcomas continue updating, it is important to examine whether, when, and which kinds of patients have had a survival improvement over the last four decades. Methods: This cohort study included 9178 patients with primary bone and joint sarcomas from 1 January 1980 to 31 December 2018 using data from Surveillance, Epidemiology and End Results (SEER)-9 Registries. The follow-up period was extended to November 2020. Patients were divided by decade into four time periods: 1980–1989, 1990–1999, 2000–2009, and 2010–2018. The primary endpoint was bone sarcomas-specific mortality (CSM). The 5-year bone sarcomas-specific survival (CSS) rate was determined stratified by demographic, neoplastic, temporal, economic, and geographic categories. The associations between time periods and CSM were examined using a multivariable Cox regression model, with reported hazard ratio (HR) and 95% confidence interval (CI). Results: The 5-year CSS rate for bone sarcomas was 58.7%, 69.9%, 71.0%, and 69.2%, in the 1980s, 1990s, 2000s, and 2010s, respectively. Older age, male gender, tumor sites at pelvic bones, sacrum, coccyx and associated joints, as well as vertebral column, osteosarcoma and Ewing tumor, and residence in non-metropolitan areas were independently associated with higher CSM risk. After adjusting for the covariates above, patients in the 1990s (HR = 0.74, 95% CI = 0.68–0.82), 2000s (HR = 0.71, 95% CI = 0.65–0.78), and 2010s (HR = 0.68, 95% CI = 0.62–0.76) had significantly lower CSM risks than patients in the 1980s. However, patients in the 2000s and 2010s did not have lower CSM risks than those in the 1990s (both *p* > 0.05). Conclusions: Although bone sarcomas survival has significantly improved since 1990, it almost halted over the next three decades. Bone sarcomas survival should improve over time, similar to common cancers. New diagnostic and therapeutic strategies such as emerging immune and targeted agents are warranted to overcome this survival stalemate.

## 1. Introduction

Primary malignant bone and joint tumors, referred to as bone sarcomas, are a set of rare neoplasms which only account for 0.2% of all malignant tumors [1]. According to a recent US population-based statistic estimate, age-adjusted rates of new cases and deaths for bone sarcomas are 1.0 and 0.5, respectively, per 100,000 people per year. Deteriorating trends include a 0.4% annual increase in new cases and a 1.3% annual increase in mortality [2]. Bone sarcomas generally include four histological types: osteosarcoma is the most common, followed by chondrosarcoma, Ewing sarcoma, and chordoma [3]. Different histological sarcomas have distinct clinical characteristics and outcomes. For instance, osteosarcoma and Ewing sarcoma often occur in pediatric patients, invade the metaphysis of long bones and cause a poor prognosis, while chondrosarcoma and chordoma often occur in middle-aged adults and leave a relatively good outcome [4,5].

Over the past few decades, with huge advances in cancer early diagnosis and treatments, survival improvements are persistently observed in many cancers [6], not only in common colorectal, breast, prostate, lung, and liver cancers [6,7,8] but also in relatively uncommon acute lymphoblastic and myeloid leukemia [9,10]. However, few studies have examined whether survival for bone sarcomas has improved. On the one hand, it is significant that advanced diagnostic imaging, surgical strategy, chemoradiotherapy, and targeted therapy have been frequently introduced in bone sarcomas practice over the years [11,12,13]. On the other hand, we must recognize that bone sarcomas as rare malignancies might still lack equal survival progress as much as common cancers. Moreover, bone sarcomas are highly heterogeneous in terms of histological type, tumor site, and onset age, all of which can hinder a homogeneous survival improvement, let alone potential racial, economic, and geographic influences. Therefore, in this large-scale retrospective cohort study, we sought to determine whether and when bone sarcomas patients have improved survival over the past four decades. Additionally, we sought to identify which patient subgroups benefited from persistent survival improvements and which did not. These efforts would largely broaden our epidemiological knowledge on bone sarcomas and help us identify a more targeted population to promote future survival.

## 2. Methods

### 2.1. Population Database

The Surveillance, Epidemiology, and End Results (SEER) Program is established by the National Cancer Institute (NCI) and has collected demographic, neoplastic, and survival data on cancer patients from population-based cancer registries since 1 January 1973 (https://seer.cancer.gov/, accessed on 10 July 2021). To gain access to the database, a data use agreement was signed. SEER*Stat software (version 8.3.9, produced by The Surveillance Research Program of the Division of Cancer Control and Population Sciences, National Cancer Institute, and Information Management Services, Inc., Calverton, NY, USA) extracted data from SEER Research Data, 9 Registries, November 2020 Submission (1975–2018). The 9 registries included San Francisco-Oakland SMSA, Connecticut, Detroit (Metropolitan), Hawaii, Iowa, New Mexico, Seattle (Puget Sound), Utah, and Atlanta (Metropolitan), covering approximately 9.4% of the US population. The SEER-9 registries program is selected because it spans the longest time period from 1975 through the current data year. The inclusion criteria were as follows: (1) tumor primary site at “Bones and Joints” based on the site recode ICD-O-3/WHO 2008; (2) malignant behavior; (3) patients diagnosed from 1 January 1980, to 31 December 2018. A total of 9351 cases were retrieved. Following that, 77 patients missing race/ethnicity and 96 patients missing survival information (autopsy/death certificate only cases) were excluded. Ultimately, 9178 patients with primary bone sarcomas across 39 years were included and analyzed. The flow diagram is presented in Supplementary Figure S1.

### 2.2. Parameters Extraction

Age was extracted as a continuous variable and further categorized into four groups: 0–19 years (children and adolescents), 20–39 years (young adults), 40–59 years (middle-aged adults), and ≥60 years (older adults). Patient sex and race were extracted. Tumor histology was extracted and categorized into five types according to ICD-O-3 recode: (1) osteosarcoma; (2) chondrosarcoma; (3) Ewing tumor; (4) chordoma; (5) other histology. The tumor primary site was extracted and categorized into seven groups: (1) long bones of lower limb and associated joints; (2) pelvic bones, sacrum, coccyx, and associated joints; (3) long bones of upper limb, scapula, and associated joints; (4) bones of skull and face and associated joints; (5) rib, sternum, clavicle, and associated joints; (6) vertebral column; (7) other sites. The year of diagnosis was extracted and categorized by decade into four time periods: (1) 1980–1989; (2) 1990–1999; (3) 2000–2009; (4) 2010–2018.

The patient economic and geographic data were only available from 1990 to 2018. Median household income was extracted and categorized into three groups: low (< USD 60,000); middle (USD 60,000–74,999); high (≥ USD 75,000) using data field “median household income inflation adjusted to 2019”. Geographic county area was categorized into three groups based on the population size: metropolitan, ≥1 million; metropolitan, <1 million; non-metropolitan, using data field “Rural-Urban Continuum Code”.

Patient vital status, cause of death, and survival months were extracted. Bone cancer-specific mortality (CSM) was defined as patient deceased due to bone sarcomas. Cancer-specific survival (CSS) was calculated as months from bone sarcomas diagnosis to CSM, which was the primary endpoint of this study.

### 2.3. Statistical Analysis

Continuous age was presented as median (quartiles) and examined by Kruskal–Wallis test among different histological types. Categorical parameters, including age, sex, race, tumor primary site, year of diagnosis, median household income, and geographic county area, were presented as number (rate) and examined by the Chi-square test among different histological types of bone sarcomas. CSS between different age, sex, race, tumor primary site, histological type, time periods, economic and geographic groups were depicted using Kaplan–Meier curves and examined using Log-rank (Mantel-Cox) test. CSS between different time periods was additionally examined using Log-rank test for trend. The 5-year CSS rates in different groups were reported and presented using a heatmap. Independent influencing factors for CSM were identified using a multivariable Cox regression model. Adjusted hazard ratio (HR), 95% confidence interval (CI), and *p*-value were reported.

A multivariable Cox regression model was also used to measure the associations between time periods and CSM in different age, sex, primary site, and histological type subpopulations. In multivariable Cox regression model 1, the decade from 1980 to 1989 was considered the reference year. In multivariable Cox regression model 2, the decade from 1990 to 1999 was considered the reference year, after excluding the decade from 1980 to 1989. The *p*-value for trends from the multivariable Cox regression model was reported. All statistical analyses and graphs were conducted using Graph-Pad Prism 7.0 (GraphPad Software Inc., San Diego, CA, USA) and SPSS 24.0 (IBM SPSS Inc., Chicago, IL, USA). A two-tailed *p* < 0.05 was considered statistically significant.

## 3. Results

### 3.1. Patients Characteristics

The patient characteristics are presented in detail in Table 1. Of 9178 bone sarcomas, 3049 (33.2%) were osteosarcoma, 2707 (29.5%) were chondrosarcoma, 1208 (13.2%) were Ewing tumor, 679 (7.4%) were chordoma, and 1535 (16.7%) were other histological types. The median onset ages of osteosarcoma, chondrosarcoma, Ewing tumor, and chordoma were 22, 52, 16, and 57 years, respectively. Ewing tumor patients were more likely to be males (61.7%). Osteosarcoma patients had a higher proportion of non-white patients (24.6%). Different histological sarcomas had different predilection sites. Osteosarcoma and chondrosarcoma usually occur in long bones of lower limb. Ewing sarcoma often occurs in long bones of lower limb and pelvis. Chordoma occurs in pelvis, skull and face bones, and vertebral column. The patient number increased along the four time periods. Patients with different histological sarcomas had similar median household incomes and geographic areas (Supplementary Table S1).

### 3.2. Risk Factors for Bone Sarcomas Mortality

In the univariable analysis, older adults (Supplementary Figure S2A), males (Supplementary Figure S2B), tumor sites at pelvic bones, sacrum, coccyx and associated joints, vertebral column, and long bones of lower limb and associated joints (supplementary Figure S2D), osteosarcoma and Ewing tumor (Supplementary Figure S2E), diagnosis in the 1980s (supplementary Figure S2F), patients with low income (Supplementary Figure S2G), and location in non-metropolitan areas (Supplementary Figure S2H) were more likely to have a lower 5-year CSS rate. White and non-white patients had similar 5-year CSS rates (Supplementary Figure S2C).

**Figure 1:**
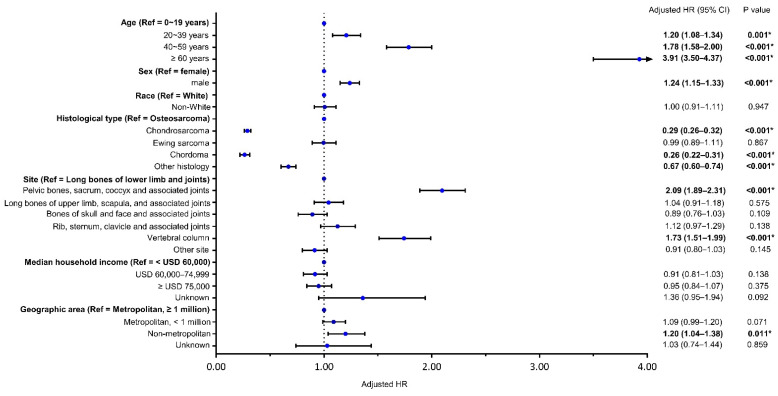
Forest map showing the independent risk factors of CSM. CSM: bone sarcomas-specific mortality; HR: hazard ratio; CI: confidence interval * Statistically significant. Bolds indicated reference variable or statistically significant.

In the multivariable analysis (Figure 1), older age, male gender, tumor sites at pelvic bones, sacrum, coccyx and associated joints, and vertebral column, and residence in non-metropolitan areas were the independent risk factors for CSM (all *p* < 0.05). Chondrosarcoma and chordoma were independent protective factors for CSM (all *p* < 0.05).

After adjusting for the variables above (Table 2), patients in the 1990s, 2000s, and 2010s had significantly lower CSM risks than those in the 1980s (all *p* < 0.05). However, patients in the 2000s and 2010s failed to have lower CSM risks than those in the 1990s (both *p* > 0.05).

### 3.3. Changes in 5-Year CSS Rates by Age, Sex, Primary Site, Histological Type, Racial, Economic, and Geographic Categories

**Figure 2:**
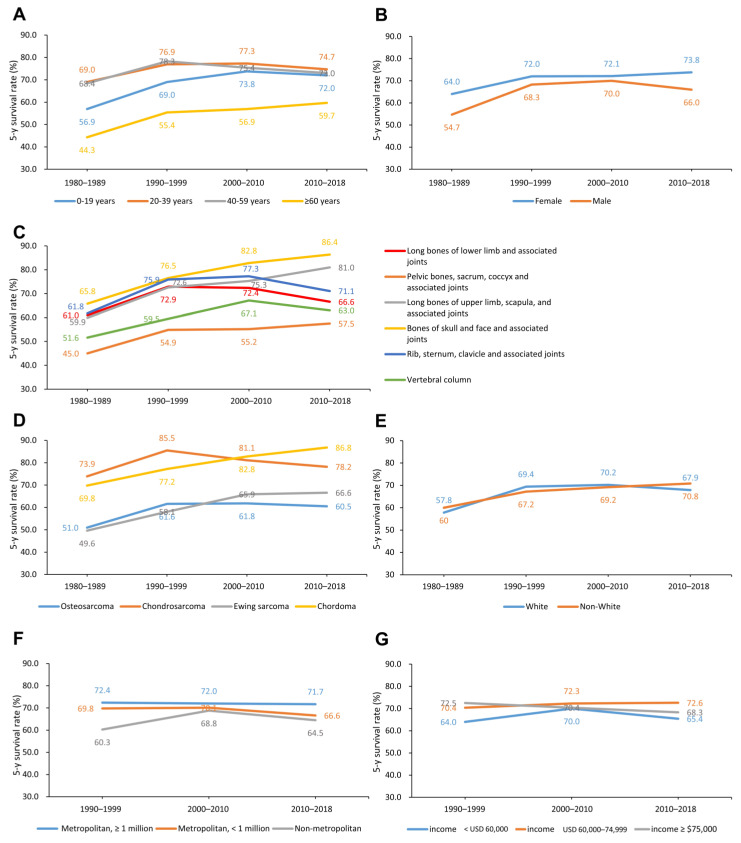
Time trends in 5-year CSS rates by decade in the categories by age (**A**), sex (**B**), primary site (**C**), histological type (**D**), racial (**E**), geographic (**F**), and economic (**G**). CSS: bone sarcomas-specific survival.

From the 1980s to 2010s, older adult patients (≥60 years) improved their 5-year survival rates by 15.4%. Children and adolescents (0–19 years) improved their 5-year survival rates by 15.1%. Young adult (20–39 years) and middle-aged adult (40–59 years) patients similarly improved their 5-year survival rates by 5.7% and 4.6%, respectively (Figure 2A). From the 1980s to 2010s, male and female patients improved their 5-year survival rates by 11.3% and 9.8%, respectively (Figure 2B).

From the 1980s to 2010s, sarcomas at long bones of upper limb, scapula, and associated joints, and bones of skull and face and associated joints similarly improved their 5-year survival rates by 21.1% and 20.6%, respectively. Sarcomas at pelvic bones, sacrum, coccyx, and associated joints and vertebral column similarly improved their 5-year survival rates by 12.5% and 11.4%, respectively. Sarcomas at rib, sternum, clavicle, and associated joints improved their 5-year survival rates by 9.3%. Sarcomas at long bones of lower limb and associated joints improved their 5-year survival rates by only 5.6% (Figure 2C).

From the 1980s to 2010s, chordoma and Ewing sarcoma accomplished the largest survival improvement by 17.0%. Osteosarcoma improved the 5-year survival rate by 9.5%. Chondrosarcoma improved the 5-year survival rate by only 4.3% (Figure 2D).

From the 1980s to 2010s, white and non-white patients similarly improved their 5-year survival rates by 10.1% and 10.8%, respectively (Figure 2E). The 5-year survival rate change was less affected by economic and geographic disparities from the 1990s to 2010s, with no absolute changes exceeding 5% (Figure 2F,G).

### 3.4. Associations between Time Periods and CSM in Specific Histological Type and Primary Site

**Figure 3:**
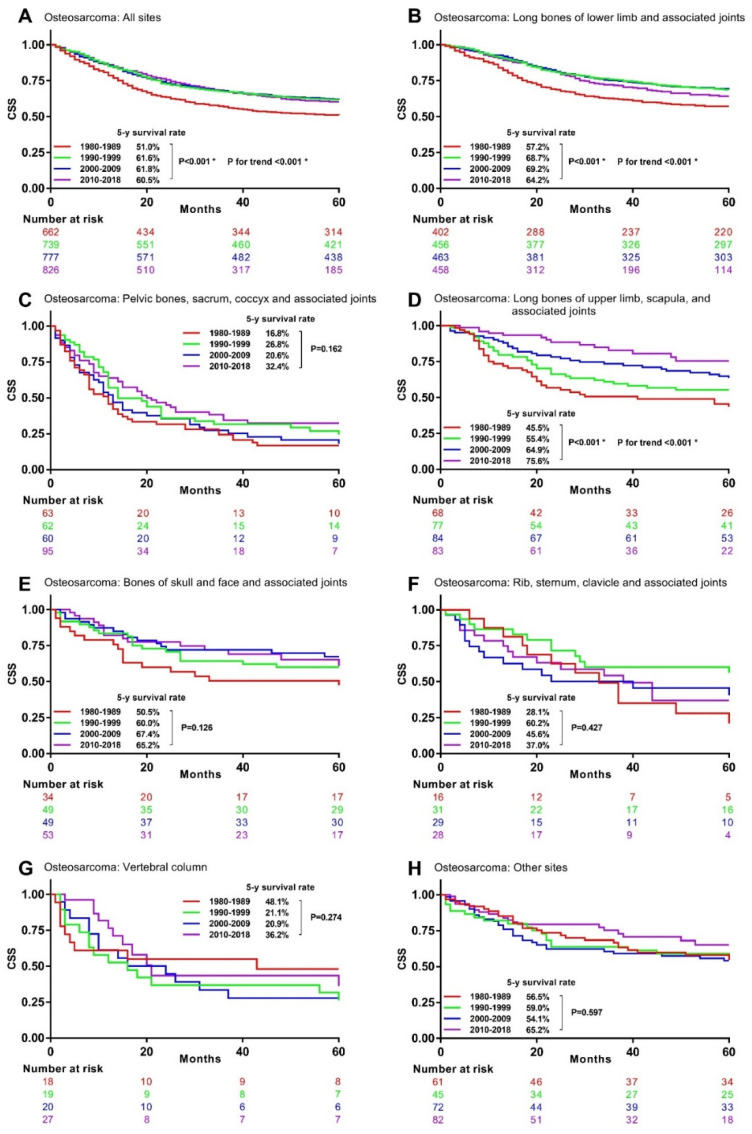
Kaplan–Meier curves showing 5-year CSS rates trend by decade in osteosarcoma with different primary sites. CSS: bone sarcomas-specific survival * Statistically significant. (**A**), All site (**B**), Long bones of lower limb and associated joints (**C**), Pelvic bones, sacrum, coccyx and associated joints(**D**), Long bones of upper limb, scapula, and associated joints (**E**), Bones of skull and face and associated joints (**F**), Rib, sternum, clavicle and associated joints (**G**), Vertebral column (**H**) Other sites.

The univariable analysis for osteosarcoma patients revealed that their 5-year CSS rates significantly increased from 51.0% in 1980s to 61.6% in the 1990s, 61.8% in the 2000s, and 60.5% in the 2010s (Figure 3A). Similar significant survival improvements were observed in osteosarcoma at long bones of lower limb and associated joints (Figure 3B), at long bones of upper limb, scapula, and associated joints (Figure 3D), but not at the other primary sites (Figure 3C,E–H).

**Figure 4:**
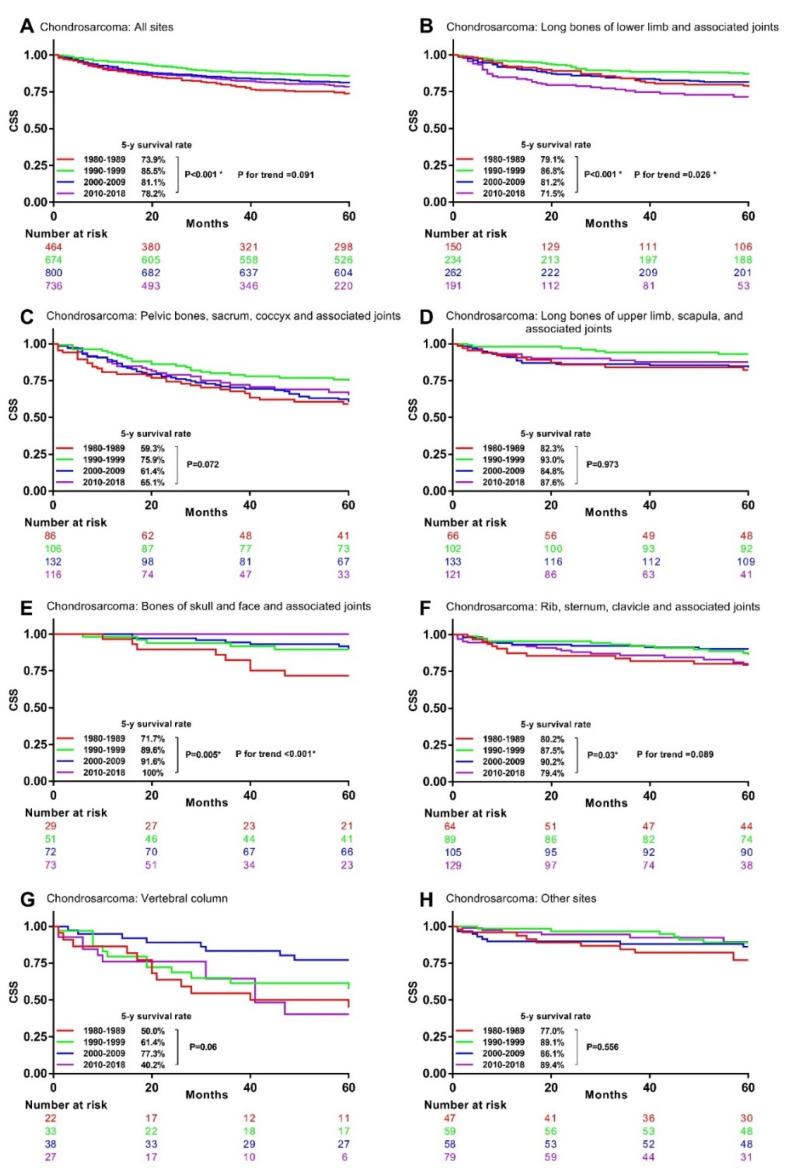
Kaplan–Meier curves showing 5-year CSS rates trend by decade in chondrosarcoma with different primary sites. CSS: bone sarcomas-specific survival. * Statistically significant. (**A**), All site (**B**), Long bones of lower limb and associated joints (**C**), Pelvic bones, sacrum, coccyx and associated joints (**D**), Long bones of upper limb, scapula, and associated joints (**E**), Bones of skull and face and associated joints (**F**), Rib, sternum, clavicle and associated joints (**G**), Vertebral column (**H**) Other sites.

The univariable analysis for chondrosarcoma patients manifested that their 5-year CSS rates significantly increased from 73.9% in the 1980s to 85.5% in the 1990s, but then decreased to 81.1% in the 2000s and 78.2% in the 2010s (Figure 4A). Significant survival changes were observed in chondrosarcoma at long bones of lower limb and associated joints (Figure 4B), bones of skull and face and associated joints (Figure 4E), rib, sternum, clavicle, and associated joints (Figure 4F), but not at the other primary sites (Figure 4C,D,G,H).

**Figure 5:**
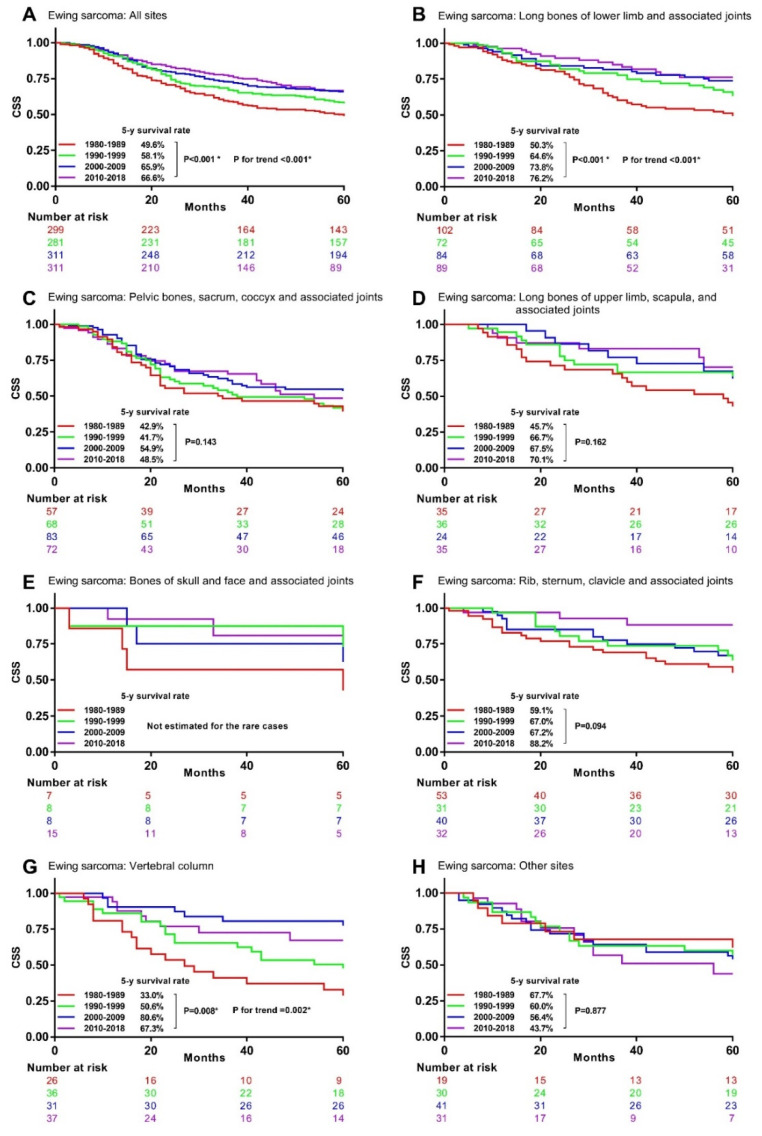
Kaplan–Meier curves showing 5-year CSS rates trend by decade in Ewing sarcoma with different primary sites. CSS: bone sarcomas-specific survival. * Statistically significant. (**A**), All site (**B**), Long bones of lower limb and associated joints (**C**), Pelvic bones, sacrum, coccyx and associated joints (**D**), Long bones of upper limb, scapula, and associated joints (**E**), Bones of skull and face and associated joints (**F**), Rib, sternum, clavicle and associated joints (**G**), Vertebral column (**H**) Other sites.

The univariable analysis for Ewing sarcoma patients indicated that their 5-year CSS rates significantly increased from 49.6% in the 1980s to 58.1% in the 1990s, 65.9% in the 2000s, and 66.6% in the 2010s (Figure 5A). Similar significant survival improvements were observed in Ewing sarcoma at long lower limb bones, associated joints (Figure 5B), and vertebral column (Figure 5G), but not at the other primary sites (Figure 5C–F,H).

**Figure 6:**
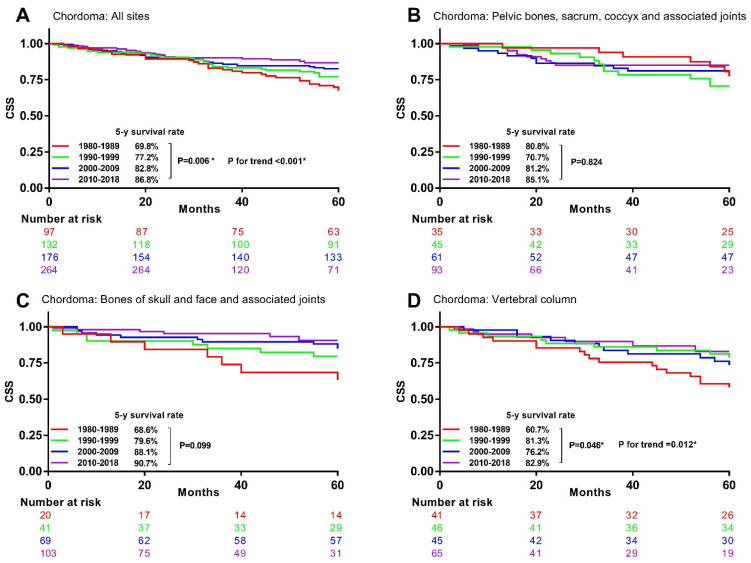
Kaplan–Meier curves showing 5-year CSS rates trend by decade in chordoma with different primary sites. CSS: bone sarcomas-specific survival. * Statistically significant. (**A**), All site (**B**), Pelvic bones, sacrum, coccyx and associated joints (**C**), Bones of skull and face and associated joints (**D**), Vertebral column.

**Figure 7:**
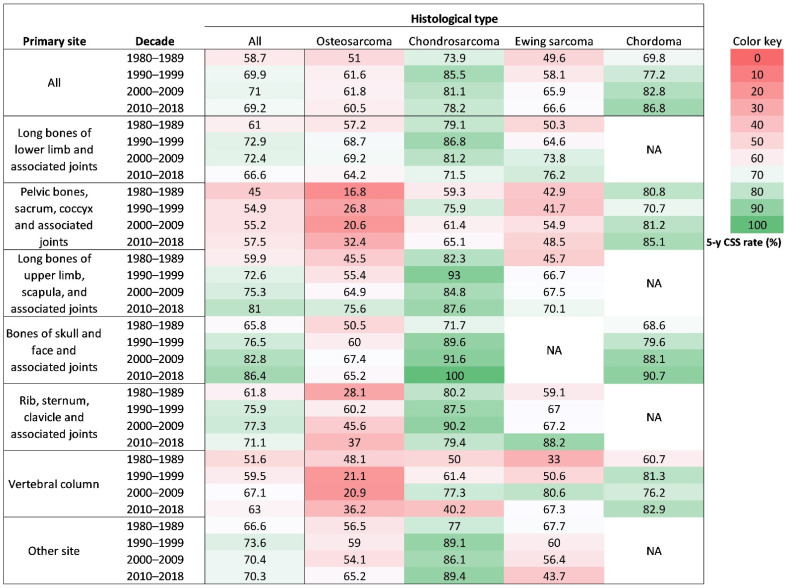
A heat map visually showing 5-year CSS rate changes in histological type and tumor site-combined categories. CSS: bone sarcomas-specific survival.

The univariable analysis for chordoma patients demonstrated that their 5-year CSS rates significantly increased from 69.8% in the 1980s to 77.2% in the 1990s, 82.8% in the 2000s, and 86.8% in the 2010s (Figure 6A). Significant survival changes were observed in chordoma at vertebral column (Figure 6D), but not at the other primary sites (Figure 6B,C). A heatmap was used to visualize the 5-year CSS rate change in specific histological and tumor site-combined categories (Figure 7).

As demonstrated in Table 3, for four histology-specific bone sarcomas, all of them displayed lower CSM risks since the 1990s than in the 1980s. Only Ewing sarcoma had a further lower CSM risk in the 2010s than in the 1990s.

As indicated in Table 4, for six site-specific bone sarcomas, except for sarcomas at long bones of the upper limb, scapula, and associated joints, and vertebral column, all of them displayed lower CSM risks since the 1990s than in the 1980s. Sarcomas at long bones of the upper limb, scapula, and associated joints, and bones of skull and face and associated joints had further lower CSM risks in the 2010s than in the 1990s.

## 4. Discussion

As primary bone sarcomas are rare cancers, they are highly unlikely to acquire equal and enough effort to improve their clinical outcome, similar to common cancers. The two largest global surveillance studies indicated that 5-year survival rate has grown steadily for most common cancers over the past three decades. However, like most large cancer epidemiological studies, the two studies did not examine how the 5-year survival rate changed for rare cancers such as bone sarcomas [14,15]. Current studies exploring bone sarcomas survival trends are relatively less. Mirabello L et al. [16] reported US population-based osteosarcoma survival between 1973 and 2004. Schneiderman et al. [17] reported US population-based chondrosarcoma survival between 1973 and 2011. These studies are confined to a certain type of bone sarcomas and are somewhat out of date. This study examined survival trends across 39 years for all bone sarcomas and four subtypes in detail over 39 years using up-to-date SEER data. We discovered that patient age, tumor histological type, and tumor site were the three most determining survival factors. In the future, additional efforts should be made to improve the survival of elderly patients, osteosarcoma and Ewing sarcoma, sarcomas at pelvis and vertebral column.

For osteosarcoma patients, the 5-year CSS rate was 51.0%, 61.6%, 61.8% and 60.5%, in the 1980s, 1990s, 2000s, and 2010s, respectively. Historically, limb amputation was the main treatment for non-metastatic osteosarcoma, with a poor 5-year survival rate of less than 20% [18,19]. Since the 1970s, chemotherapy advancements such as Adriamycin, cis-platin, and methotrexate have increased the prevalence of limb salvage surgery and elevated the 5-year survival rate to more than 50% [20]. However, our study demonstrated that current diagnosis and treatment modalities for osteosarcomas stopped further improvement of the 5-year CSS rate after the 1990s, leaving it at a poor 5-year CSS rate at about 61%. Therefore, more randomized controlled trials (RCTs) are required to identify new effective agents for osteosarcoma.

First, with regard to chemotherapy for osteosarcoma, many RCTs obtained a non-ideal result in recent years. For example, zoledronate displays potent anti-osteosarcoma properties via multiple cellular molecular mechanisms in preclinical studies [21]. The French Sarcoma Group conducted the large-scale open-label phase 3 trial OS2006 in 318 patients with newly diagnosed high-grade osteosarcoma. The patients were randomized to receive standard chemotherapy without or with zoledronate (158 vs. 160). OS2006 results showed that addition of zoledronate does not reduce the treatment failure risk and is even detrimental [22]. Another large-scale phase 3 international trial EURAMOS-1 enrolled 618 resectable high-grade osteosarcoma patients: 310 received postoperative cisplatin, doxorubicin, and methotrexate (MAP) and 308 received MAP plus ifosfamide and etoposide (MAPIE). EURAMOS-1 results showed that addition of ifosfamide and etoposide to postoperative chemotherapy does not improve event-free survival and even increases toxic reaction. Enhanced chemotherapy seems to delay osteosarcoma metastasis but cannot be transformed into patient’s survival benefit [23]. MAP regimen is still the standard treatment for osteosarcoma patients. It remains a major challenge to further enhance the chemotherapy efficacy for osteosarcoma based on the classical MAP regimen. Secondly, with regard to radiotherapy for osteosarcoma, promisingly, the simple 2D-palliative radiotherapy has gradually transformed into a 3D-comformal radiotherapy since 2010 [24]. Radiotherapy is effective in some of the selected osteosarcoma patients. In addition, alpha emitter radium-223 can be a new radiation option for metastatic osteosarcoma but is just reported in some clinical cases [25,26]. It still takes a long time to develop radiotherapy to generally improve the survival of osteosarcoma patients. Thirdly, targeted agents are generally unsatisfactory because osteosarcoma carries few targetable mutations. In the future, osteosarcoma may be treated by enhancing immunotherapy using agents such as mifamurtide [27,28,29]. Mifamurtide can activate tumor-associated macrophages and kill the sarcoma cells. Mifamurtide is the first drug to improve long-term survival of osteosarcoma patients over the past 20 years. However, currently, mifamurtide is mainly applicable to non-metastatic resectable osteosarcoma [30,31].

For chondrosarcoma patients, the 5-year CSS rate was 73.9%, 85.5%, 81.1% and 78.2%, in the 1980s, 1990s, 2000s, and 2010s, respectively. Our study demonstrated that despite chondrosarcoma overall having a good survival, chondrosarcoma at spinal column and pelvis continued to have a poor prognosis. Unlike osteosarcoma and Ewing sarcoma which predispose to children and adolescents, chondrosarcoma usually occurs in elderly patients and is an aging-related sarcoma [32]. Houdek MT and colleagues [33] indicated that advancing patient age is associated with worse survival and disease recurrence in pelvic chondrosarcoma. With the national population ageing during the last few decades, chondrosarcoma patients tend to have an increasing onset age. This might be one of the reasons for a declining survival rate of chondrosarcoma over time. In addition, with chondrosarcoma patient number increasing by years, lack of diagnosis and treatment standardization such as extended surgery may also contribute to a declining survival rate. Primary tumor resection enhances chondrosarcoma survival regardless of whether distant metastasis occurs [34,35]. On the other hand, for unresectable chondrosarcoma, carbon ion radiotherapy can be a new treatment option [36,37]. However, all these new therapeutic strategies are recently identified and need time to test. Amer et al. [38] advocated for chondrosarcoma subtyping because clinical characteristics and survival significantly differed between subtypes such as myxoid, juxtacortical, clear-cell, mesenchymal, and dedifferentiated. Targeted therapies such as isocitrate dehydrogenase (IDH) mutation are promising in chondrosarcoma and are expected to improve survival in the future [39,40].

In our study, Ewing sarcoma and chordoma had the greatest survival improvement by 17.0% from the 1980s to 2010s. Ewing sarcoma is a highly invasive small round cell tumor sensitive to chemotherapy and radiotherapy [41,42]. Our study revealed that Ewing sarcoma at pelvis had the worst survival. Much work has recently been aimed at improving survival of pelvic Ewing’s sarcoma. Radiotherapy has been demonstrated to increase survival in patients with surgically treated pelvic Ewing’s sarcoma [43,44]. Unlike osteosarcoma chemotherapy trials, Ewing sarcoma chemotherapy trials have conferred significant survival benefits on patients over the past few decades. In 1990, the first Intergroup Ewing’s Sarcoma Study (IESS) proved that VAC plus ADR regimen (vincristine, actinomycin D, cyclophosphamide, and Adriamycin) is significantly superior to VAC regimen alone, with the 5-year survival rates of 65% and 28%, respectively [45]. In 2012, NCT00006734 trial found that, for localized Ewing sarcoma patients receiving VDC-IE regimen, chemotherapy every 2 weeks is superior to chemotherapy every 3 weeks, with the 5-year progression-free survival rates of 73% and 65%, respectively [46]. Our data demonstrated that Ewing sarcoma patients continue to lower CSM risk not only in the 1990s but also in the 2010s, indicating that those chemotherapy trials’ success may produce a real-world benefit in the population. Notably, lung recurrence acts as a fatal hidden killer of Ewing sarcoma. Recently, the R2Pulm trial demonstrated that, for Ewing sarcoma patient with lung metastasis, autologous stem-cell rescue has no benefit when compared to conventional chemotherapy and whole-lung irradiation [47]. Close imaging follow-up to monitor recurrence is expected to further improve Ewing sarcoma survival [48]. In our study, chordoma revealed a good 5-year CSS rate and survival improvement even in pelvis and spinal column, which might benefit from advancements in surgical technique and radiotherapy [49]. A high local recurrence rate is a characteristic of chordoma [50]. Recent progress of concurrent radiotherapy and targeted therapy (imatinib or erlotinib) might help further improve advanced or recurrent chordoma survival [51,52].

There are inevitably limitations in our study. We did not observe the survival changes by different tumor TNM stages. This study spanned a long time period from 1980 to 2018. Many patients lacked the TNM staging information during such a long time period. The American Joint Committee on Cancer (AJCC) TNM staging criteria for bone sarcomas have also changed several versions during this period and might not be suitable to merge. In addition, bone sarcomas are often staged by the Enneking staging system, which deserves to be used in the SEER program in the future.

## 5. Conclusions

In summary, over the past three decades, bone sarcomas survival did not continue to significantly progress, particularly for osteosarcoma and chondrosarcoma. Pelvic and spinal sarcomas continue to impose treatment challenges. Besides traditional surgical resection, chemotherapy, and radiotherapy progress, emerging targeted and immune therapy should be actively developed for bone sarcomas. Bone sarcomas survival should improve over time, similar to common cancers.

## Figures and Tables

**Table 1:** Demographic and neoplastic characteristics of the patients, 1980–2018.

Parameter	Overall (*n* = 9178)	Osteosarcoma (*n* = 3049)	Chondrosarcoma (*n* = 2707)	Ewing sarcoma (*n* = 1208)	Chordoma (*n* = 679)	Other (*n* = 1535)
Age (years)						
Median (quartiles)	39 (18–61)	22 (14–49)	52 (38–66)	16 (12–23)	57 (42–70)	54 (32–71)
0–19 years	2522 (27.5%)	1390 (45.6%)	115 (4.2%)	813 (67.3%)	24 (3.5%)	180 (11.7%)
20–39 years	2070 (22.6%)	682 (22.4%)	625 (23.1%)	308 (25.5%)	125 (18.4%)	330 (21.5%)
40–59 years	2058 (22.4%)	431 (14.1%)	962 (35.5%)	65 (5.4%)	222 (32.7%)	378 (24.6%)
≥60 years	2528 (27.5%)	546 (17.9%)	1005 (37.1%)	22 (1.8%)	308 (45.4%)	647 (42.1%)
Sex						
Female	4033 (43.9%)	1351 (44.3%)	1259 (46.5%)	463 (38.3%)	282 (41.5%)	678 (44.2%)
Male	5145 (56.1%)	1698 (55.7%)	1448 (53.5%)	745 (61.7%)	397 (58.5%)	857 (55.8%)
Race						
White	7571 (82.5%)	2300 (75.4%)	2374 (87.7%)	1094 (90.6%)	578 (85.1%)	1225 (79.8%)
Non-white	1607 (17.5%)	749 (24.6%)	333 (12.3%)	114 (9.4%)	101 (14.9%)	310 (20.2%)
Tumor primary site						
Long bones of lower limb and associated joints	3456 (37.7%)	1793 (58.8%)	842 (31.1%)	348 (28.8%)	1 (0.1%)	472 (30.7%)
Pelvic bones, sacrum, coccyx and associated joints	1489 (16.2%)	292 (9.6%)	448 (16.5%)	280 (23.2%)	240 (35.3%)	229 (14.9%)
Long bones of upper limb, scapula, and associated joints	995 (10.8%)	313 (10.3%)	425 (15.7%)	130 (10.8%)	0 (0.0%)	127 (8.3%)
Bones of skull and face and associated joints	857 (9.3%)	187 (6.1%)	227 (8.4%)	39 (3.2%)	236 (34.8%)	168 (10.9%)
Rib, sternum, clavicle and associated joints	714 (7.8%)	107 (3.5%)	393 (14.5%)	158 (13.1%)	1 (0.1%)	55 (3.6%)
Vertebral column	670 (7.3%)	86 (2.8%)	124 (4.6%)	131 (10.8%)	198 (29.2%)	131 (8.5%)
Other site	997 (10.9%)	271 (8.9%)	248 (9.2%)	122 (10.1%)	3 (0.4%)	353 (23.0%)
Year of diagnosis						
1980–1989	1841 (20.1%)	674 22.1%)	469 (17.3%)	301 (24.9%)	100 (14.7%)	297 (19.3%)
1990–1999	2225 (24.2%)	750 (24.6%)	681 (25.2%)	281 (23.3%)	132 (19.4%)	381 (24.8%)
2000–2009	2525 (27.5%)	786 (25.8%)	806 (29.8%)	314 (26.0%)	178 (26.2%)	441 (28.7%)
2010–2018	2587 (28.2%)	839 (27.5%)	751 (27.7%)	312 (25.8%)	269 (39.6%)	416 (27.1%)

**Table 2:** Trends in the adjusted HR for CSM.

Decade	1980–1989	1990–1999		2000–2009		2010–2018		
Model		Adjusted HR (95% CI)	*p*	Adjusted HR (95% CI)	*p*	Adjusted HR (95% CI)	*p*	*p* for Trend
Multivariable model 1	1.00	0.74 (0.68–0.82)	<0.001 *	0.71 (0.65–0.78)	<0.001 *	0.68 (0.62–0.76)	<0.001 *	<0.001 *
Multivariable model 2	-	1.00		0.96 (0.87–1.05)	0.365	0.93 (0.84–1.04)	0.190	0.184

In the multivariable model 1, the 1980s was considered as the reference year. Adjusted HR was calculated after controlling for patient’s age, sex, race, histological type, and tumor site. In the multivariable model 2, the 1990s was considered as the reference year. Adjusted HR was calculated after controlling for patient’s age, sex, race, histological type, tumor site, median household income and geographic county area. * Statistically significant. CSM: bone sarcomas-specific mortality HR: hazard ratio CI: confidence interval.

**Table 3:** Trends in the adjusted HR for CSM in specific histological types.

Decade	1980–1989	1990–1999		2000–2009		2010–2018		
Histological Type		Adjusted HR (95% CI)	*p*	Adjusted HR (95% CI)	*p*	Adjusted HR (95% CI)	*p*	*p* for Trend
**Multivariable model 1**								
Osteosarcoma	1.00	0.75 (0.64–0.87)	<0.001 *	0.74 (0.63–0.86)	<0.001 *	0.65 (0.56–0.77)	<0.001 *	<0.001 *
Chondrosarcoma	1.00	0.68 (0.55–0.85)	0.001 *	0.77 (0.62–0.95)	0.016 *	0.88 (0.70–1.12)	0.300	0.478
Ewing sarcoma	1.00	0.63 (0.50–0.79)	<0.001 *	0.53 (0.42–0.67)	<0.001 *	0.48 (0.37–0.63)	<0.001 *	<0.001 *
Chordoma	1.00	0.85 (0.59–1.22)	0.366	0.65 (0.45–0.95)	0.026 *	0.58 (0.37–0.90)	0.015 *	0.005 *
**Multivariable model 2**								
Osteosarcoma	_	1.00		0.97 (0.83–1.14)	0.712	0.88 (0.74–1.05)	0.149	0.153
Chondrosarcoma	_	1.00		1.13 (0.91–1.40)	0.287	1.34 (1.05–1.71)	0.019 *	0.021 *
Ewing sarcoma	_	1.00		0.83 (0.65–1.06)	0.140	0.74 (0.56–0.99)	0.041 *	0.035 *
Chordoma	_	1.00		0.85 (0.58–1.25)	0.409	0.75 (0.47–1.19)	0.220	0.208

In the multivariable model 1, the 1980s was considered as the reference year. Adjusted HR was calculated after controlling for patient’s age, sex, race, and tumor site. In the multivariable model 2, the 1990s was considered as the reference year. Adjusted HR was calculated after controlling for patient’s age, sex, race, tumor site, median household income, and geographic county area. * Statistically significant. CSM: bone and joint sarcomas-specific mortality HR: hazard ratio CI: confidence interval.

**Table 4:** Trends in the adjusted HR for bone sarcomas-specific mortality (CSM) in specific tumor sites.

Decade	1980–1989	1990–1999		2000–2009		2010–2018		
Primary Site		Adjusted HR	*p*	Adjusted HR	*p*	Adjusted HR	*p*	*p* for Trend
**Multivariable model 1**								
Long bones of lower limb and associated joints	1.00	0.68 (0.58–0.79)	<0.001 *	0.70 (0.60–0.82)	<0.001 *	0.80 (0.68–0.95)	0.01 *	0.006 *
Pelvic bones, sacrum, coccyx and associated joints	1.00	0.76 (0.62–0.94)	0.011 *	0.81 (0.66–0.99)	0.043 *	0.65 (0.52–0.81)	<0.001 *	0.001*
Long bones of upper limb, scapula, and associated joints	1.00	0.80 (0.59–1.07)	0.128	0.68 (0.50–0.93)	0.016 *	0.51 (0.35–0.74)	<0.001 *	<0.001 *
Bones of skull and face and associated joints	1.00	0.65 (0.45–0.94)	0.021 *	0.43 (0.29–0.62)	<0.001 *	0.32 (0.21–0.51)	<0.001 *	<0.001 *
Rib, sternum, clavicle and associated joints	1.00	0.68 (0.48–0.97)	0.033 *	0.59 (0.41–0.86)	0.006 *	0.79 (0.54–1.16)	0.226	0.122
Vertebral column	1.00	0.87 (0.64–1.19)	0.393	0.68 (0.49–0.94)	0.019 *	0.66 (0.46–0.94)	0.022 *	0.006 *
**Multivariable model 2**								
Long bones of lower limb and associated joints	_	1.00		1.05 (0.89–1.24)	0.561	1.22 (1.02–1.46)	0.032 *	0.036 *
Pelvic bones, sacrum, coccyx and associated joints	_	1.00		1.10 (0.90–1.36)	0.351	0.88 (0.70–1.11)	0.282	0.298
Long bones of upper limb, scapula, and associated joints	_	1.00		0.87 (0.63–1.19)	0.384	0.65 (0.44–0.95)	0.027 *	0.029 *
Bones of skull and face and associated joints	_	1.00		0.63 (0.44–0.90)	0.012 *	0.48 (0.31–0.75)	0.001 *	0.001 *
Rib, sternum, clavicle and associated joints	_	1.00		0.84 (0.57–1.25)	0.396	1.16 (0.77–1.74)	0.483	0.534
Vertebral column	_	1.00		0.78 (0.56–1.09)	0.147	0.76 (0.53–1.09)	0.137	0.121

In the multivariable model 1, the 1980s was considered as the reference year. Adjusted HR was calculated after controlling for patient’s age, sex, race, and histological type. In the multivariable model 2, the 1990s was considered as the reference year. Adjusted HR was calculated after controlling for patient’s age, sex, race, histological type, median household income, and geographic county area. * Statistically significant. CSM: bone sarcomas-specific mortality; HR: hazard ratio; CI: confidence interval.

## Data Availability

All data were extracted from the Surveillance, Epidemiology, and End Results Program (https://seer.cancer.gov/, accessed on 10 July 2021).

## Supplementary Materials

The following are available online at https://www.mdpi.com/article/10.3390/cancers13215381/s1, Figure S1: Flowchart showing patient selection., Figure S2: Kaplan-Meier curves showing 5-year CSS rates stratified by demographic, neoplastic, temporal, economic and geographic characteristics, Table S1: Economic and geographic characteristics of the patients, 1990–2018.
