# Supplementary material for: Trends in Tumor Site-Specific Survival of Bone Sarcomas from 1980 to 2018: A Surveillance, Epidemiology and End Results-Based Study
Source: Cancers (Basel). 2021 Oct 27;13(21):5381. doi: 10.3390/cancers13215381 (PMC8582558; doi:10.3390/cancers13215381)
Supplement: Supplementary file 1 [file cancers-13-05381-s001.zip › cancers-1405001-supplementary/supplementary, proofed/Supplementary Figure S2.pdf]

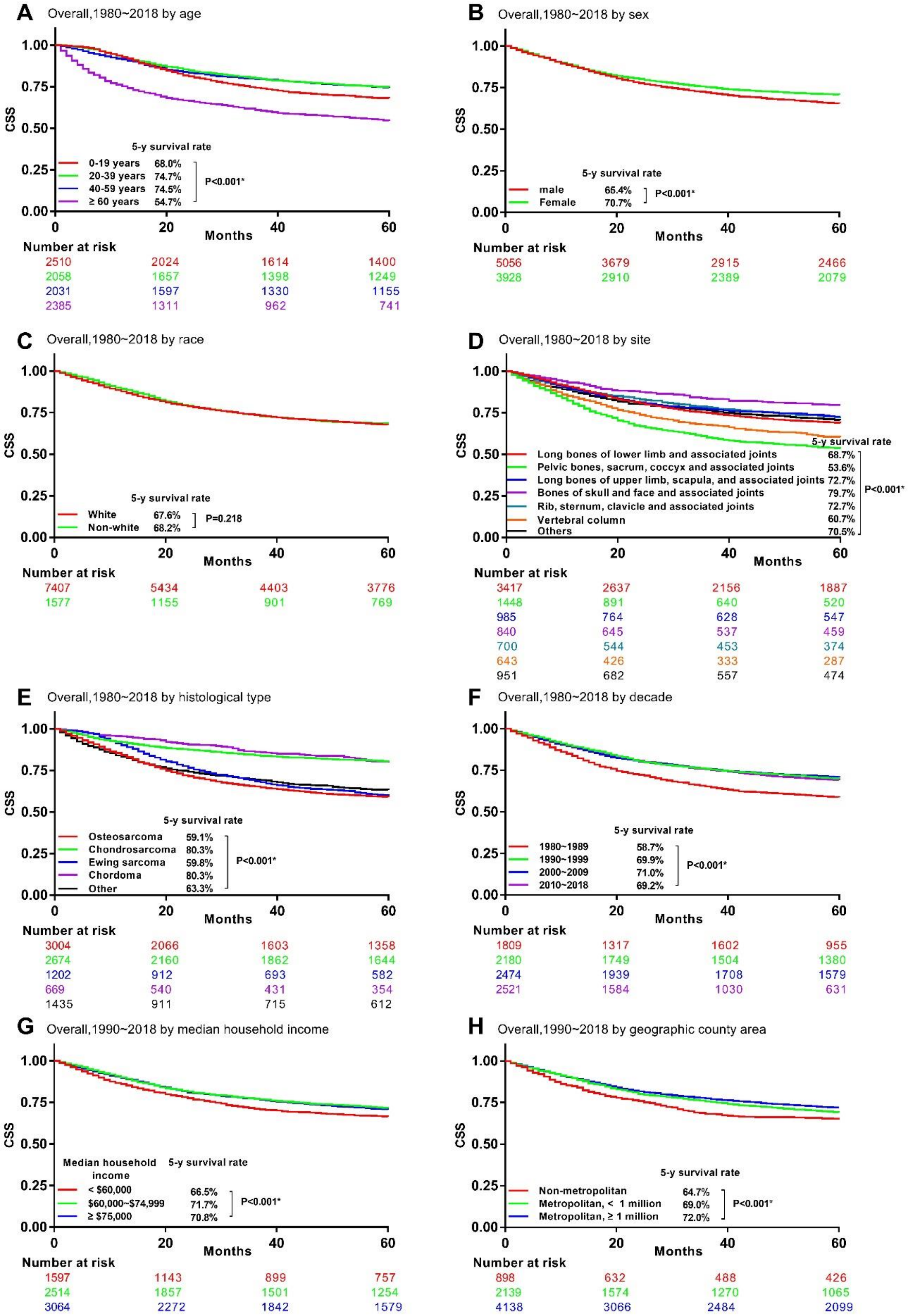

Supplementary Figure S2. Kaplan-Meier curves showing 5-year CSS rates stratified by demographic (**A,B,C**), neoplastic (**D,E**), temporal (**F**), economic (**G**) and geographic characteristics (**H**). CSS: bone sarcomas-specific survival * Statistically significant.
