# Supplementary material for: Trends in Tumor Site-Specific Survival of Bone Sarcomas from 1980 to 2018: A Surveillance, Epidemiology and End Results-Based Study
Source: Cancers (Basel). 2021 Oct 27;13(21):5381. doi: 10.3390/cancers13215381 (PMC8582558; doi:10.3390/cancers13215381)
Supplement: Supplementary file 1 [file cancers-13-05381-s001.zip › cancers-1405001-supplementary/supplementary, proofed/Supplementary Table S1.pdf]

Supplementary Table S1. Economic and geographic characteristics of the patients, 1990–2018.

Parameter	Overall (n=7337)	Osteosarcoma (n=2375)	Chondrosarcoma (n=2238)	Ewing sarcoma (n=907)	Chordoma (n=579)	Other (n=1238)
Median household income						
< USD 60,000	1639 (22.3%)	537 (22.6%)	504 (22.5%)	216 (23.8%)	114 (19.7%)	268 (21.6%)
USD 60,000-74,999	2572 (35.1%)	841 (35.4%)	788 (35.2%)	311 (34.3%)	190 (32.8%)	442 (35.7%)
≥ USD 75,000	3126 (42.6%)	997 (42.0%)	946 (42.3%)	380 (41.9%)	275 (47.5%)	528 (42.6%)
Geographic area, population size						
Metropolitan, ≥ 1 million	4136 (56.4%)	1368 (57.6%)	1241 (55.5%)	479 (52.8%)	340 (58.7%)	708 (57.2%)
Metropolitan, < 1 million	2187 (29.8%)	682 (28.7%)	693 (31.0%)	290 (32.0%)	172 (29.7%)	350 (28.3%)
Non-metropolitan	919 (12.5%)	285 (12.0%)	285 (12.7%)	130 (14.3%)	59 (10.2%)	160 (12.9%)
Unknown	95 (1.3%)	40 (1.7%)	19 (0.8%)	8 (0.9%)	8 (1.4%)	20 (1.6%)
