# Supplementary figures and images for: Trends in Tumor Site-Specific Survival of Bone Sarcomas from 1980 to 2018: A Surveillance, Epidemiology and End Results-Based Study
Source: Cancers (Basel). 2021 Oct 27;13(21):5381. doi: 10.3390/cancers13215381 (PMC8582558; doi:10.3390/cancers13215381)

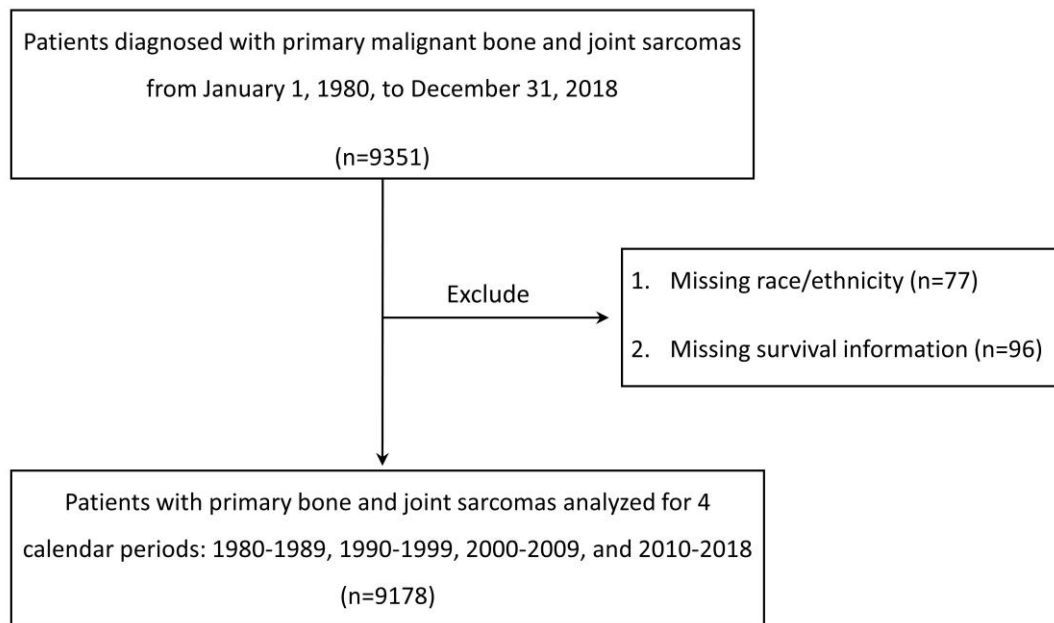

**Supplementary Figure S1.** Flowchart showing patient selection.

Supplement: Supplementary file 1 [file cancers-13-05381-s001.zip › cancers-1405001-supplementary/supplementary, proofed/Supplementary Figure S1.pdf]
